# Occupational stress among medical residents in educational hospitals

**DOI:** 10.1186/s40557-018-0262-8

**Published:** 2018-08-08

**Authors:** Sedigheh Ebrahimi, Zahra Kargar

**Affiliations:** Department of Medical Ethics, Shiraz University of Medical Sciences, Shiraz, Iran

**Keywords:** Occupation, Resident, Stress

## Abstract

**Background:**

Occupational stress and its related psychological strain is a concern among resident doctors that may affect patient care adversely. Residents face many stresses because of their high job demands in delivery of hospital care. They are often subject to work load and pressure due to direct involvement with patients, prolonged working hours, poor job opportunities and low support. Their multiple educational and clinical roles can also affect their performance and quality of personal or professional life. The aim of this study was to evaluate the occupational stress among residents of various medical specialties.

We aimed to explore the reasons of occupational stress in residents’ life and determine how we can enhance the stress-coping strategies and create more suitable conditions.

**Methods:**

This cross-sectional analytical-descriptive study was conducted on all medical residents with various specialties in Shiraz University of Medical Sciences. Data was collected using Osipow occupational stress questionnaire and analyzed by SPSS software version 17.

**Results:**

The response rate was 88.8%. The average stress score of all residents was 156.35 out of 250. The highest and lowest average stress scores belonged to gynecology and dermatology specialties, respectively. The highest average score of the stress factors was related to the workload with the score of 35.09 of 50 (moderate to severe stress). The total stress score had a significant relationship with age (*P* = 0.030) and sex (*P* = 0.009) as well as lack of time to get the needed healthy meals (*P* = 0.047), high work hours (*P* < 0.01), surgical specialties (*P* < 0.01) and on call shift (*P* < 0.01).

**Conclusion:**

Since most of the stressors were related to the workload, interventions such workload reduction, education about occupational stress and its management, promoting interpersonal relations and more supportive measures are recommended.

## Background

Stress is known as a biological and psychological process experienced by a person in encountering the environmental threats. Occupational stress, is the biological and psychological effects of negative interaction between work conditions and person’s knowledge, skills, or expectations [1]. It occurs when there is no coordination between responsibilities and pressures and personal abilities, characteristics and needs, inhibiting one’s ability to cope. Occupational stress can lead to poor health and even individual damage [1].

There are a variety of stressors in medical workplace. In the meantime, residents face with a lot of stress because of the workload and heavy duties as well as a large volume of scientific literature and practical tasks which must be learnt in a limited time [2, 3]. In addition, there are a number of issues identified as being stressful for residents, such as financial problems and low income, being evaluated without enough training, and being under psychological and physical pressure from both their superiors and patients. Stress can also be the result of any workplace stressors which are related to role characteristics including role conflicts (such as the conflict between duty to supervisors, or attending physicians and patients expectation), role ambiguities (the tasks were not defined well) and role overload (too many tasks and daily work load for the available time, and other constraints in fulfilling heavy duty expected from them and responsibility for patients’ health [1, 4, 5]. So, depression and anxiety during residency is expected and are certainly effective on the quality of patient’s care [6].

Results of studies in Iran which were conducted to evaluate the occupational stress in different fields of medicine and in medical residents, showed moderate to high level of stress. Workload, responsibility, exam stress, financial difficulties and high work hours per week and less rest time were considered as the effective factors to create stress in residents [1, 7]. However, there were no study conducted in our region evaluating the ideas of residents themselves mentioning the stressors of their working environment and any ideas for providing a better workplace for them. The present study evaluates the effective factors leading to occupational stress in residents of educational hospitals affiliated to Shiraz University of medical sciences. The objective of this study was to identify the reasons of stress in medical residents. This helps promote the policy programs and providing practical solutions to reduce stress in the workplace of residents. Indeed, creating more suitable condition may improve the quality of patients’ care.

## Methods

This cross-sectional analytical-descriptive study was performed in 2015. The study population included medical residents with various medical specialties in Shiraz University of Medical Sciences, here 315 out of 350 residents. The inclusion criteria were absence of any chronic physical disease and who having spent their first 6 months of the residency.

The Osipow standard questionnaire is a measurement instrument for determining the occupational stress level of the study population. The information collected via a modified version of questionnaire which was congruent with the area of research [1, 8].

The validity and reliability (Cronbach-Alphas coefficient more than 0.85) of this version of the questionnaire was approved through several national studies in Iran [1].

This questionnaire used the 5 dimensions of workload, role inadequacy, role ambiguity, role conflict and responsibility to evaluate stress and its related psychological strain. Each dimension consists of 10 questions in a 5-points Likert scale (as “never” = 1 to “most of the time”=5). Demographic information about the respondents were collected and compared to stress scores.

Based on the obtained scores, the effect of each stressor was classified in 4 categories of scores: mild (10 to 19), moderate (20 to 29), high (30 to 39) and severe (40 to 50). The total stress was also classified in 4 categories of scores: mild (50 to 99), moderate (100 to 149), high (150 to 199) and severe (200 to 250), respectively.

Data were collected and analyzed using SPSS 17.0 software. To evaluate the relationship between variables and occupational stress level, one-way ANOVA- Fisher test, t-test, and correlation coefficient were used. Statistical significance was considered as 0.05 or less.

## Results

**Fig. 1:**
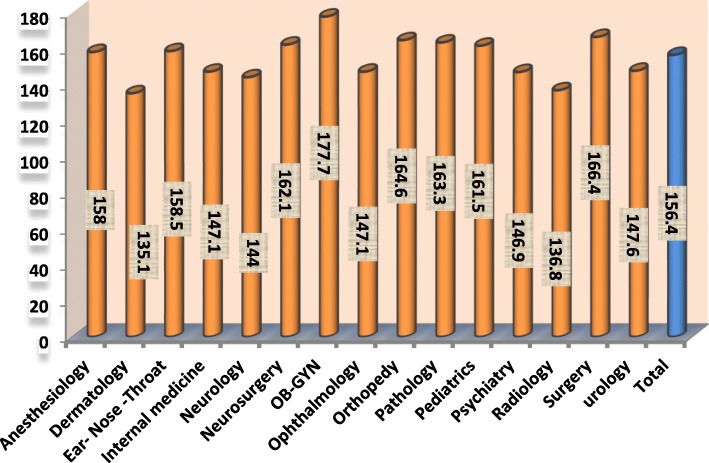
The average score of stress of residents in various specialties

Totally, 311 out of 350 questionnaires which were given to the residents were completed. (Response rate = 88.8%).The average score of total stress in residents in various specialties were shown in Fig. 1. The results showed that the average score of residents’ total stress was 156.35 which was considered as high. The highest (177.72) and lowest (135.08) average scores of stress belonged to Gynecology (*N* = 37) and dermatology residents (*N* = 12), respectively. 57.8% of residents had a high level and 42.2% of them had a moderate level of occupational stress.

The relationship between total stress and each effective factor on it, was evaluated in each case according to t-test outcome (Table 1).

**Table 1:** The relation between total stress and each of effective factors on it

Effective factor		Descriptive Statistics			*P* value
		N (%)	Mean score of total stress	SD	
Rotation in specialty	rotating	189 (60.8%)	156.5	18.2	0.832
	non- rotating	122 (39.2%)	156.1	18.8	
Hours of rest during the on-call shifts	< 2 h	83 (27.76)	157.7	16.7	0.428
	≥ 2 h	228 (73.3%)	155.8	19.0	
Time to eat healthy meals	enough	142 (45.7%)	155.2	18.9	0.047
	not enough	169 (54.3%)	158.9	17.3	
Specialty fields	Surgical	164 (52.7%)	162.7	18.4	< 0.001
	Internal medical	147 (47.3%)	149.2	15.6	
Having on-call shift	On-call	295 (94.9%)	157.5	18.0	< 0.001
	No on-call	16 (5.1%)	134.8	10.6	
Hours of work per week	≥ 80 h	182 (82%)	158.6	17.9	< 0.001
	< 80 h	56 (18%)	145.9	17.0	

In Table 2, the average of total stress was shown according to demographic variables in residents. Total stress was increased with age. It was higher in female residents than males. Marital status, having children and smoking had no significant relationship with total stress.

**Table 2:** The average of total occupational stress according to the baseline characteristics of residents (*N* = 311)

(Baseline characteristics of residents) Variables		Frequency (percent)	Average of total stress	*P* value
Age	< 31 year	118 (37.9%)	153.85	0.03
	≥ 31 years	193 (62.1%)	157.87	
Gender	Female	140 (45%)	153.87	0.009
	Male	171 (55%)	159.37	
Marriage	Single	125 (40.2%)	157.68	0.30
	Married	186 (59.8%)	155.45	
Having children	With child	79 (25.4%)	154.65	0.345
	No child	232 (74.6%)	156.92	
Smoking	smoker	35 (11.3%)	155,94	0.890
	nonsmoker	276 (88.7%)	156.40	

In Table 3, The Means, Standard deviations and correlations between residency post-graduate Year and total stress (PGY), were shown.

**Table 3:** The Correlations between the total stress and Residency Post Graduate Year (PGY)

Parameters Statistical Index PGY	Number (percent)	Mean total stress(SD)	Minimum	Maximum
PGY-1	88 (28.3)	151.1 (13.2)	123	182
PGY-2	97 (31.2)	156.2 (17.7)	111	197
PGY-3	72 (23.2)	158.7 (19.2)	121	198
PGY-4	54 (17.4)	162.1 (23.5)	109	198
Total	311 (100)	156.4 (18.3)	109	198

One-way ANOVA has calculated a mean total stress score for each of the four PGY group of residents. Then, Comparing between the mean of total stress in four groups by Fisher test showed significant differences (*P* = 0.003).

A pair-wise comparison using the post-hoc Tukey’s test to find different groups showed significant differences in total stress between the first and third years (*P* = 0.041) and also between the first and fourth year residents (*p* = 0.003). There are no significant differences in total stress among the others (*P* > 0.05). By arranging the first and second year residents as juniors (*N* = 185, Mean score = 153.7 ± 15.8) and third and fourth year residents as senior residents (*N* = 126, Mean score = 160.2 ± 21.1), T-test showed a significant difference between the total stress score in these two groups (*P* = 0.002).

The factors of workload (*P* = 0.003) and role inadequacy (*P* = 0.036) were significantly higher in junior residents, in contrast to the responsibility factor (*P* = 0.000) which was higher significantly in senior residents. There were no significant differences in both junior and senior residents in terms of role ambiguity (*P* = 0.255) and role conflict (*P* = 0.107).

In Table 4, the frequency and severity of each sub-category of occupational stressors compared with the total stress affecting the residents were evaluated based on level of stress scores.

**Table 4:** The average of total stress according to the Stressor sub-categories affecting residents

Stressor sub-categories	Level of stress against individual stressor domain					Average score stress
	Mild (10 to 50)	Moderate (20 to 29)	High (30 to 39)	Severe (40 to 50)	Total	
Frequency (%)	N(%)	N(%)	N(%)	N(%)	N(%)	
Workload	3 (1)	57 (18.3)	166 (53.4)	85 (27.3)	311 (100)	35.09
Role-inadequacy	0 (0)	110 (35.4)	197 (63.3)	4 (1.3)	311 (100)	31.14
Role ambiguity	9 (2.9)	145 (46.6)	151 (48.6)	6 (1.9)	311 (100)	29.51
Role conflict	4 (1.3)	154 (49.5)	149 (47.9)	4 (1.3)	311 (100)	29.71
Responsibility	19 (6.1)	130 (41.8)	106 (34.1)	56 (18)	311 (100)	30.87
Total	0 (0)	120 (38.6)	191 (61.4)	0 (0)	311 (100)	156.35

Table 4 shows that the average level of stress due to workload was higher than the other stressors in different fields of residency (35.09). The responsibility and role inadequacy stressors were also high and both role ambiguity and role conflict were in moderate level.

In general, the average of total stress of the residents (156.35) was in high level according to classification mentioned previously. Internal correlation between the total stress and sub-categories of occupational stressors were determined using the Pearson’s correlation coefficient.

The significant positive linear relationship was found between all dimensions of occupational stressors, except for two variables of responsibility and role ambiguity (*P* > 0.05). The highest positive linear relationship was between role conflict and role ambiguity (*r* = 0.476, *p* < 0.01).

According to the correlation analysis, high positive linear correlation was between the total stress and role conflict (*r* = 0.792), workload (*r* = 0.697), role ambiguity (*r* = 0.627) and role inadequacy (*r* = 0.536) sub-categories (all *p*-values were smaller than 0.001).

## Discussion

Many of the residents in the present study had a high (57.8%) to moderate (42.2%) occupational stress. This result is consistent with study by Bahreinian et al. in Iran which showed that73.4% of physicians tolerate a mild to severe stress with 75.5% and 65% in residents and specialists, respectively. Examination and learning stress and then economic problems were more important stressors in residents [7]. Malek et al. reported a moderate stress level in most of the residents with the highest average score related to the workload and responsibility. Stress levels of residents having on call shifts, surgery, more working hours per week or with less resting time within duty were significantly higher [1]. Some studies in other countries also suggested that workload is one of the most important risk factors for stress [9, 10]. This may be due to very high performance expectations, high working hours and workload in residency programs. This shows that we should pay greater attention to workload planning and practical policies to reduce fatigue and workload intensity during resident training programs.

In our study, a significant positive relationship was found between the age and occupational stress (*P* = 0.030). In studies of Torrado Oubina et al. and Bahreinian et al. no significant relationship between age groups and occupational stress was found [7, 11].

In contrast, in a study by Shimiizo in Japan, the highest stress rates were found in the youngest group [12]. According to Osipow et al., life stage will reflect differences in occupational stresses and will result in differing availability of coping resources [13].

Also, our study found a significant relationship between gender and occupational stress (*P* = 0.009). This result corroborated previous studies which were conducted in Canada and Japan, Their results showed that, occupational stress in females was more than men [12, 14] and this may be due to greater involvement of females from male colleagues in responsibility for household chores and social engagement combining with work roles.

Against our study, in two other studies on the residents, stress was more in male than female residents [15, 16]. In another National Survey by Malek et al., there was no statistically significant difference between males and females [1].

No significant relationship was found in our study regarding marital status and stress (*p* = 0.297) which was consistent with other similar studies [1, 11]. In contrast, in a study in by Danayifakhr et al., psychological problems were more in married ones [17] and in another study, single residents experienced higher level of stress compared to the married ones [18]. Also, our research exhibited no considerable relationship significant relationship (*P* = 0.345) regarding having children and stress level which was consistent with Malek et al. [1].

We found no significant relationship in regards to circulating hospitals in residency (*P* = 0.832) and having enough time to rest within duty (*P* = 0.428). Total stress in the group without enough time for healthy meals within duty was more than the group who had enough time.

In case of specialties which require night on-call shift, there was also a significant difference between this variable and total stress, in current study (*P* < 0.001). Other studies showed similar results indicating that detrimental effects of having night duty on total stress [1].

Having night duty causes less time to rest and leisure and spending less time with the family which can increase the person’s stress and upset his mental balance.

Also, our study showed a significant relationship between the surgical specialty and stress. Same results were obtained from the other study [19]. it may be due to greater exposure of this group with critically ill, emergency and high mortality risk patients.

Our results showed that the residents who worked more than 80 h per week, had higher stress compared to the others. In a study on orthopedics residents, prolong working hours was known as the most important reason of residents’ stress [9]. In accordance with the recommendations of Occupational Safety and Health Association (OSHA) in 2012, working hours of all residency courses should be limited to 80 h per week [20].

Our findings showed that junior residents (first and second years) are more likely to have work stress. Similarly, study by Alexander et al. determined that there was a significant difference regarding the stress of physical environment, self-care and personal string in the first-year residents compared to their attending physicians [21]. The reasons behind these findings may be lack of experience to use coping resources, lack of orientation to academic and clinical activities, *lack* of support by team members,dealing with their superiors, staff and patients.

In a study conducted by Schneider et al. in Texas on a group of gynecology residents, no significant difference was observed in residents with different years of education [2].

As a limitation of this study, it should be mentioned that alcohol consumption was omitted in our questionnaire since it was prohibited for Muslim societies in Iran.

## Conclusion

Occupational stress affects residents’ quality of life negatively, which in turn can influence on patients health care and outcomes. According to the results of the current and previous studies, it is essential to pay more attention to residents ‘stress.

Stress can be decreased by reducing the workload and working hours, particularly first and second year of residency. It can be moderated by supportive measures, particularly as workload levels increase in high postgraduate years of residency.
